# Parathyroid hormone 1 receptor signaling mediates breast cancer metastasis to bone in mice

**DOI:** 10.1172/jci.insight.157390

**Published:** 2023-03-08

**Authors:** Srilatha Swami, Hui Zhu, Aria Nisco, Takaharu Kimura, Matthew J. Kim, Vaisakh Nair, Joy Y. Wu

**Affiliations:** Department of Medicine, Division of Endocrinology, Stanford University School of Medicine, Stanford, California, USA.

**Keywords:** Bone Biology, Oncology, Breast cancer, G protein&ndash;coupled receptors, Mouse models

## Abstract

Bone metastases are a common complication of breast cancer. We have demonstrated that intermittent administration of parathyroid hormone (PTH[1-34]) reduces the incidence of bone metastases in murine models of breast cancer by acting on osteoblasts to alter the bone microenvironment. Here, we examined the role of signaling mediated by PTH 1 receptor (PTH1R) in both osteoblasts and breast cancer cells in influencing bone metastases. In mice with impaired PTH1R signaling in osteoblasts, intermittent PTH did not reduce bone metastasis. Intermittent PTH also did not reduce bone metastasis when expression of PTH1R was knocked down in 4T1 murine breast cancer cells by shRNA. In 4T1 breast cancer cells, PTH decreased expression of PTH-related protein (PTHrP), implicated in the vicious cycle of bone metastases. Knockdown of PTHrP in 4T1 cells significantly reduced migration toward MC3T3-E1 osteoblasts, and migration was further inhibited by treatment with intermittent PTH. Conversely, overexpression of PTHrP in 4T1 cells increased migration toward MC3T3-E1 osteoblasts, and this was not inhibited by PTH. In conclusion, PTH1R expression is crucial in both osteoblasts and breast cancer cells for PTH to reduce bone metastases, and in breast cancer cells, this may be mediated in part by suppression of PTHrP.

## Graphical abstract

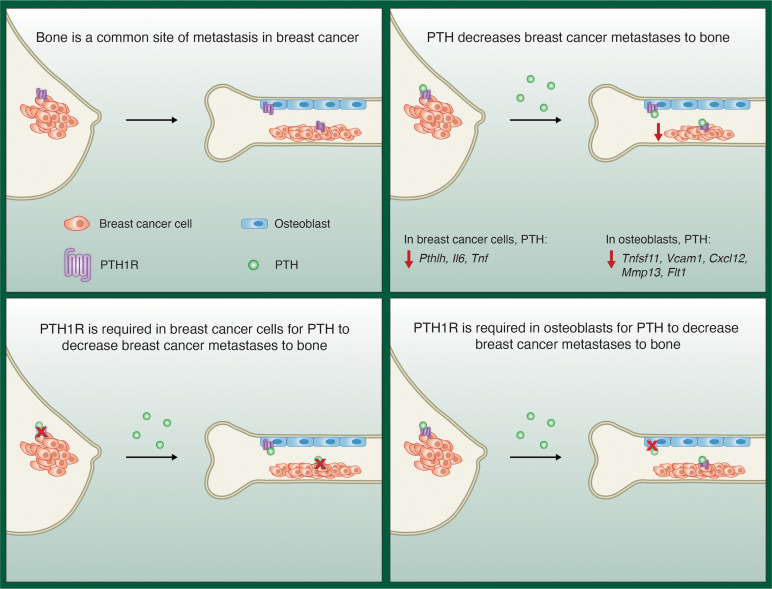

## Introduction

Breast cancer is the most frequently diagnosed cancer in women and continues to be a leading cause of death in the United States and worldwide (1). Advanced breast cancer with distant organ metastasis is incurable, and bone is the most common site of metastasis (2, 3). Bone metastases are characterized by osteolytic lesions and can cause bone loss, pain, fracture, hypercalcemia, and other skeletal adverse events (4). Although breast cancer therapies are effective and focus mostly on the tumor cells, there is a need for agents that target the skeletal-related events and restore bone integrity.

Bone metastasis depends on the ability of the cancer cells to home to and engraft in the bone. Parathyroid hormone–related protein (PTHrP; encoded by *Pthlh)* is produced by breast cancer cells and increases RANKL (encoded by *Tnfsf11*) in the bone while reducing osteoprotegrin (OPG; encoded by *Tnfrsf11b*), thereby shifting the RANKL to OPG ratio to establish a vicious cycle in favor of increased bone resorption (5). The vicious cycle is further perpetuated by the release into the bone milieu of growth factors present in the bone matrix, such as TGF-β, IGFs, BMPs, and MMPs (5). These factors promote homing, invasion, colonization, survival, and proliferation of breast cancer cells within bone, leading to increased bone resorption and bone loss.

Antiresorptive drugs such as bisphosphonates and denosumab (an mAb targeted against the osteoclast differentiation factor RANKL) are highly effective in reducing the adverse skeletal events related to bone metastases (6). However, antiresorptive medications do not support the new bone formation needed to restore skeletal integrity, and high cumulative doses are associated with osteonecrosis of the jaw and atypical fractures (7, 8). Hence, strategies that preserve bone health and restore bone loss are an attractive proposition.

Osteoporosis is a chronic condition characterized by an imbalance between bone formation and resorption leading to loss of bone volume, decreased bone strength, and increased fracture risk (9). Anabolic therapies for osteoporosis increase bone formation by increasing proliferation and/or differentiation of osteoblasts. Currently approved anabolic drugs for treatment of osteoporosis include parathyroid hormone type 1 receptor (PTH1R) ligands teriparatide (parathyroid hormone [PTH(1-34)]) (10) and abaloparatide (a synthetic analog of PTHrP[1-34]) (11), and romosozumab, an mAb directed against the Wnt inhibitor sclerostin (12, 13). Data on the safety of anabolic therapies for treatment of skeletal metastasis, however, are limited. Studies targeting cancer and bone metastasis have reported that altering the bone microenvironment with intermittent PTH decreases tumor engraftment in multiple myeloma and myelogenous leukemia but not in acute myeloid leukemia (14, 15). Studies have also shown that intermittent PTH treatment increases the osteoblastic niche and metastatic prostate cells in mice (16). Preclinical studies from our laboratory have demonstrated that anabolic PTH decreases both the tumor engraftment rate and the incidence of spontaneous breast cancer metastases to bone in orthotopic models of both human and murine breast cancer tumors and prolongs survival in mice (17). Mechanistically, intermittent PTH treatment altered the metastatic gene expression profile in MC3T3-E1 osteoblastic cells and attenuated the migratory potential of breast cancer cells towards osteoblasts.

The biological actions of both PTH and PTHrP are mediated by the G protein–coupled PTH1R (18, 19). PTH1R is expressed in many tissues that also express PTHrP, including breast cancer cells (20–22).The anabolic actions of PTH on PTH1R are mediated by the stimulatory G protein Gs, which activates adenylyl cyclase, leading to an increase in cAMP levels and the activation of the PKA pathway (23, 24). Although the main physiological function of PTH signaling involves the regulation of calcium homeostasis, PTHrP functions more as a multifunctional cytokine (25), and its role in breast cancer and metastasis has been well studied (26–28). We have shown that although PTH had no effect on the growth of the breast cancer cells or primary tumors, PTH altered the gene expression profile of the primary tumor (17).

In this study, we examined the role of PTH1R signaling in both breast cancer cells and osteoblasts in influencing breast cancer skeletal metastases in response to intermittent PTH. Using shRNA and transgenic models with impaired PTH1R signaling, we demonstrate that PTH1R is required in both breast cancer cells and osteoblasts for PTH to reduce breast cancer bone metastases in mice.

## Results

### Loss of PTH1R signaling in osteoblasts increases migration of 4T1 breast cancer cells in vitro.

**Figure 1:**
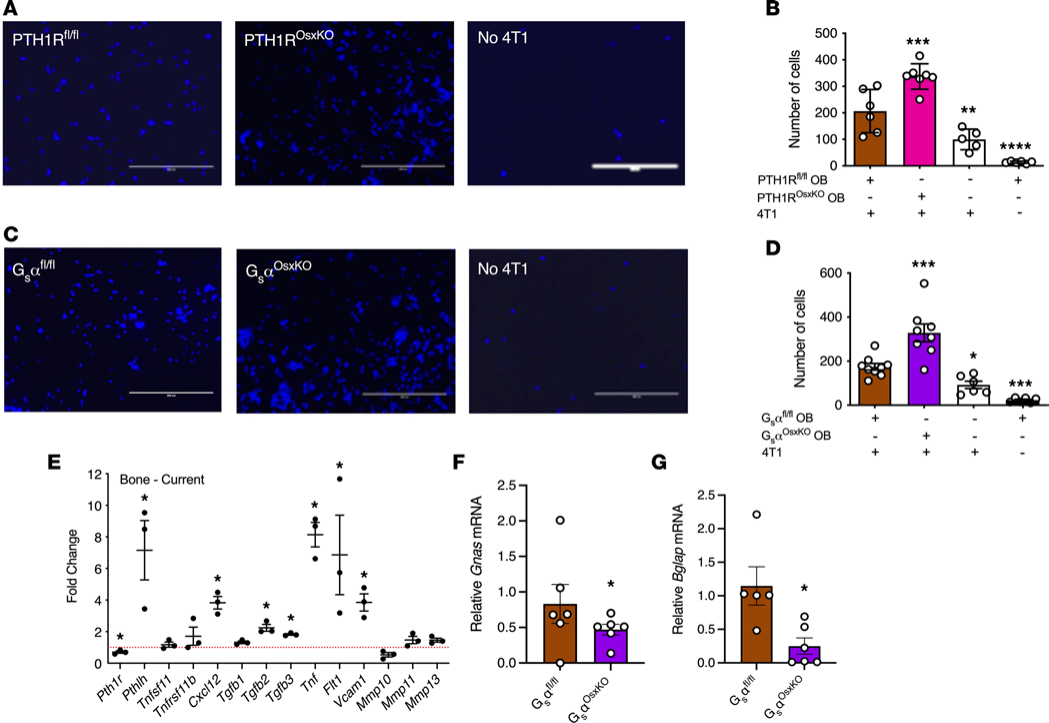
Loss of PTH1R signaling in calvarial cells enhances migration of 4T1 cells in vitro and alters expression of target genes involved in the breast–bone vicious cycle. To study the effects of PTH1R knockdown in osteoblasts (OB), primary osteoblasts were isolated from neonatal calvariae of PTH1R^OsxKO^ and G_s_α^OsxKO^ mice, and migration assays were set up against 4T1 cells in transwell chambers. (**A**) Representative images and (**B**) numbers of 4T1 breast cancer cells that migrated toward osteoblasts from PTH1R^OsxKO^ mice vs control (PTH1R^fl/fl^) mice. All values represent the mean ± SEM of 6 individual experiments conducted in triplicate. Statistical significance was evaluated using 2-way ANOVA with Tukey’s test as the post hoc analysis. Scale bar: 200 μm. (**C**) Representative images and (**D**) numbers of 4T1 breast cancer cells that migrated toward osteoblasts from G_s_α^OsxKO^ vs control (G_s_α^fl/fl^) mice. All values represent the mean ± SEM of 6 individual experiments conducted in triplicate. Statistical significance was evaluated using 2-way ANOVA with Tukey’s test as the post hoc analysis. Scale bar: 200 μm. (**E**) Target gene expression in bone from PTH1R^OsxKO^ mice (*n* = 3) compared with PTH1R^fl/fl^ (*n* = 3) using RNA-Seq analysis as described in Methods. The red dotted line represents expression in PTH1R^fl/fl^ mice set to 1. (**F**) Gene expression of *Gnas* (the gene encoding G_s_α) and (**G**) *Bglap* (the gene encoding osteocalcin) in forelimbs of control and G_s_α^OsxKO^ mice that were administered doxycycline in utero until weaning. All values represent the mean ± SEM. Statistical significance was evaluated using 1-way ANOVA with Tukey’s test as the post hoc analysis. *P < 0.05, **P < 0.01, ***P < 0.001, and ****P < 0.0001.

To determine whether migration of 4T1 breast cancer cells toward osteoblasts is affected by PTH1R signaling, primary calvarial osteoblasts were isolated from neonatal mice with a conditional ablation of either PTH1R (PTH1R^OsxKO^ mice) or the Gs α subunit (G_s_α) (G_s_α^OsxKO^ mice) in osteoprogenitors, and cocultured with 4T1 cells in a transwell migration assay system. We plated 4T1 cells in the transwell inserts in serum-free medium, and they were allowed to migrate toward calvarial osteoblasts plated in the bottom chamber. Migration of 4T1 breast cancer cells was increased in the presence of primary calvarial osteoblasts from PTH1R control (PTH1R^fl/fl^) or G_s_α control (G_s_α^fl/fl^) mice, as compared with wells with serum-containing medium only (Figure 1, A–D). Migration of 4T1 breast cancer cells was further increased toward primary calvarial osteoblasts lacking either PTH1R or G_s_α, suggesting that PTH1R signaling in osteoblasts negatively regulates secreted promigratory factors acting on breast cancer cells.

We recently performed RNA-Seq on osteoprogenitors from bones of PTH1R^fl/fl^ and PTH1R^OsxKO^ mice (29). We examined the expression pattern of several genes implicated in the breast–bone vicious cycle in the bone microenvironment and found that conditional ablation of PTH1R in osteoblasts is associated with increased expression of *Pthlh*, *Cxcl12*, *Tgfb2*, *Tgfb3*, *Tnf, Flt1* (encoding VEGFR1), and *Vcam1* (Figure 1E). Osteoblasts from PTH1R^OsxKO^ mice had significantly lower levels of *Pth1r* mRNA. Gene expression analysis revealed that the expression of *Gnas*, which encodes G_s_α, was significantly lower in the bones of G_s_α^OsxKO^ mice (Figure 1F). *Gnas* mRNA is still present at a low level because of ubiquitous expression of G_s_α within non–Cre-targeted cells. We have previously demonstrated that conditional ablation of G_s_α in osteoprogenitors disrupts PTH1R signaling (24). Consistent with this, the expression of PTH target osteocalcin (encoded by *Bglap*) is significantly decreased in G_s_α^OsxKO^ bone (Figure 1G). These changes suggest that the lack of PTH1R or disruption of its signaling pathways significantly alters the bone microenvironment in both the PTH1R^OsxKO^ and G_s_α^OsxKO^ mice.

### Ablation of G_s_α, a downstream mediator of PTH1R signaling in bone, abolishes the inhibitory effects of PTH treatment on breast cancer bone metastasis.

To study the role of PTH1R signaling in breast cancer bone metastasis, we attempted to generate PTH1R^OsxKO^ mice that survive to adulthood. In both PTH1R^OsxKO^ and G_s_α^OsxKO^ mice, constitutive expression of Osx-driven Cre recombinase throughout embryonic development results in early death (30–32). However, the osterix promoter–driven GFP:Cre fusion protein is regulated by a Tet-Off tetracycline transactivator (33), and administration of doxycycline in drinking water to pregnant females from mating until delivery can delay Cre recombinase expression until after birth (24). We backcrossed PTH1R^OsxKO^ mice to a Balb/c background to provide a syngeneic background for the 4T1 tumor injections. Unfortunately, PTH1R^OsxKO^ mice on the Balb/c background could not be rescued by doxycycline-mediated delay of PTH1R deletion until postnatal life. We have previously shown that in the G_s_α^OsxKO^ mice, the anabolic actions of PTH on bone are mediated by Gs (24). Hence, we have used G_s_α^OsxKO^ mice backcrossed onto a Balb/c background for our studies.

**Figure 2:**
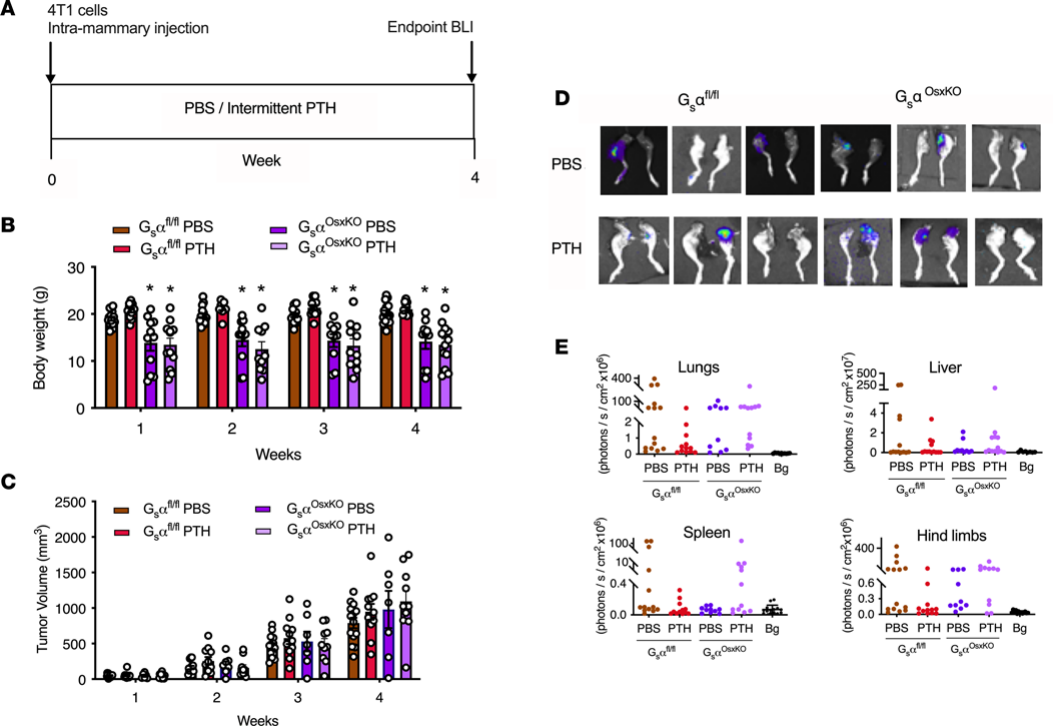
Ablation of G_s_α, a downstream target of PTH1R signaling in bone, abolishes the beneficial effects of PTH treatment on skeletal metastasis with breast cancer. (**A**) Experimental design. Mice were 10 weeks old at the time of 4T1 cell injection. PBS or intermittent PTH (80 mg/kg) was administered Monday through Friday. (**B**) Weekly BW measurements and (**C**) weekly tumor volume measurements in control (G_s_α^fl/fl^) and G_s_α^OsxKO^ mice treated with PBS or PTH. (**D**) Representative bioluminescent images of metastases to hind limbs in G_s_α^fl/fl^ and G_s_α^OsxKO^ mice treated with PBS or PTH (80 mg/kg/d, Monday through Friday) (**E**) Quantification of BLI in lungs, liver, spleen, and hind limbs with metastases. All values represent the mean ± SEM of 10 mice for each group. Statistical significance was evaluated using 2-way ANOVA with Tukey’s test as the post hoc analysis. *P < 0.05 when compared with respective control. Bg, background.

We injected 4T1 breast cancer cells into the mammary fat pads of 10-week-old control (G_s_α^fl/fl^) and G_s_α^OsxKO^ mice. PTH treatment was initiated 24 hours later (Figure 2A), and mice were monitored for the next 4 weeks. G_s_α^OsxKO^ mice were significantly smaller than their control littermates throughout the study period, and PTH treatment did not affect the body weights of either control or G_s_α^OsxKO^ mice (Figure 2B). Palpable tumors were detected in all groups by week 1, and tumor volumes were similar in all 4 groups, reaching approximately 800 to 1000 mm^3^ (Figure 2C) by the end of the treatment period. PTH treatment did not affect the growth of the tumors in G_s_α^OsxKO^ mice or G_s_α^fl/fl^ littermate controls. Mice were euthanized 4 weeks after tumor implantation, and individual organs were dissected and underwent bioluminescence imaging (BLI) to detect presence of metastases (Supplemental Figure 1; supplemental material available online with this article; https://doi.org/10.1172/jci.insight.157390DS1). We confirmed that bioluminescence correlated with metastasis by histological analysis of paraffin-embedded sections from G_s_α^OsxKO^ mice (Supplemental Figure 2). Metastases to the lungs, liver, and spleen were similar in both the PBS-treated control and G_s_α^OsxKO^ mice, and intermittent PTH had no additional effect (Table 1 and Figure 2, D and E). As expected, PTH treatment significantly reduced bone metastases to the hind limbs in control mice (*n* = 3 of 12) compared with the PBS-treated control mice (*n* = 8 of 14). However, in G_s_α^OsxKO^ mice, PTH administration had no effect on bone metastases, suggesting that PTH1R signaling is required in osteoblasts for PTH to decrease bone metastasis.

### Decreased expression of Pth1r is associated with progression and metastasis of breast cancer in both mice and humans.

**Figure 3:**
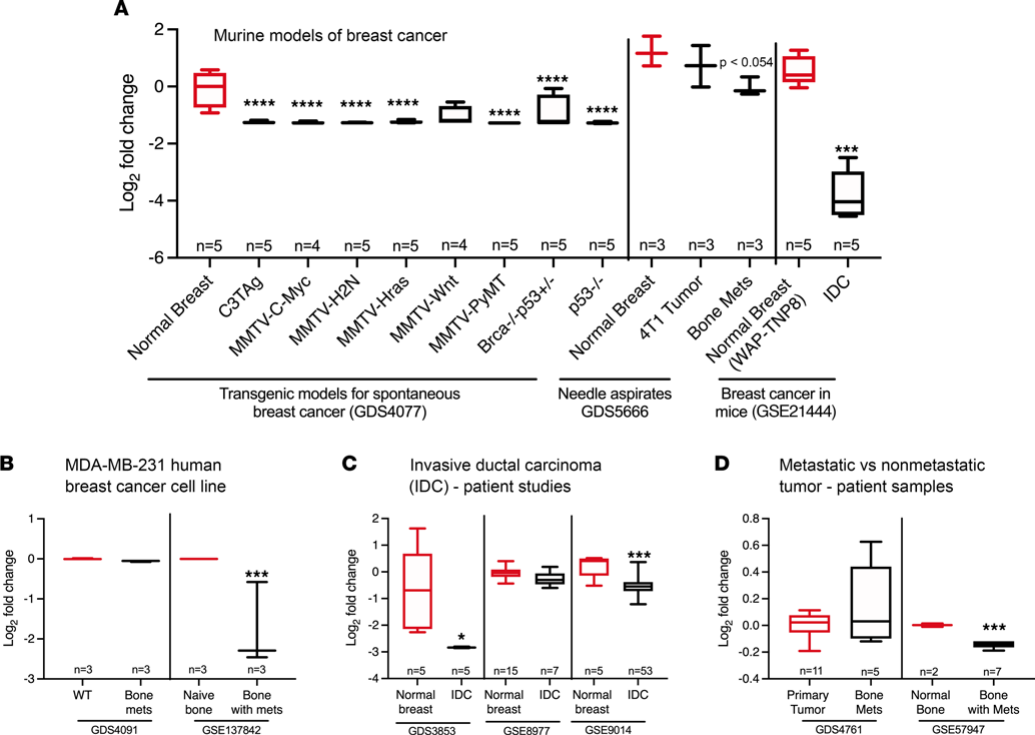
Microarray analyses of publicly available data sets for the expression of *Pth1r* in breast cancer. Publicly analyzed microarray data sets from (**A**) murine breast cancer studies, (**B**) human breast cancer cell lines, and (**C** and **D**) studies of human patients with breast cancer were analyzed for the expression of *Pth1r* in murine and human breast cancer tissues using the GEO2R (an online data analysis tool used to analyze GEO data sets under the same experimental conditions) as described in Methods. Values are represented as log_2_ fold change in comparison to their respective controls, as indicated. *P < 0.05, ***P < 0.001, ****P < 0.0001 when compared to their respective controls. Mets, metastasis.

PTH1R is also expressed by breast cancer cells (20, 22). To examine the relationship between PTH1R expression and breast cancer progression, we analyzed publicly available microarray data sets in the National Center for Biotechnology Information (NCBI) database. A total of 10 data sets using murine models of breast cancer, human cell lines, or patient samples were analyzed for PTH1R expression. All 3 murine studies analyzed showed significant decreases in *Pth1r* expression in breast cancer samples compared with normal breast tissue (Figure 3A). In the first study (34) , using 8 different genetically engineered murine models of spontaneous breast cancer, *Pth1r* was significantly lower in the breast tumor tissue compared with normal mammary gland from pregnant mice on mixed background (Figure 3A). In needle aspirates of bone metastases (35), *Pth1r* expression was decreased compared with the 4T1-derived primary tumor. In another murine study (36), involving transgenic mice that synthesize the large T antigen under the control of the whey acidic protein promoter–TNP8 mice resulting in multifocal ductal carcinoma in situ and, ultimately, invasive ductal breast carcinoma (IDC), *Pth1r* levels were significantly lower in IDC than in breast tissue from WT nonlactating mice (Figure 3A).

Results from the 7 analyzed human studies were more varied (Figure 3, B–D), with 4 studies (37–39) reporting reduced levels of *PTH1R* expression and 3 studies (40–42) reporting no change. *PTH1R* expression in the bone variant MDA-MB-231 human breast cancer cell line, characterized by highly metastatic clones that preferentially migrate to the bone, did not differ from the parental cell line in 1 study. In a second study using the same MDA-MB-231 human breast cancer cell line, *PTH1R* expression was significantly decreased in cells from bone metastases compared with naive bone (Figure 3B). In 2 of the 3 studies involving patients with IDC, *PTH1R* expression was significantly lower in cancerous tissue compared with adjacent, normal, healthy breast stroma (Figure 3C). In 2 studies of patients with bone metastases, *PTH1R* expression in 1 study was significantly lower in bone with metastases compared with normal bone, but *PTH1R* expression did not differ between bone metastases and the primary tumor in the other study (Figure 3D). In no instance was an increase in *Pth1r/PTH1R* expression associated with progression of disease. More importantly, we did not find any cases in which reduced PTH1R expression was associated with a better clinical outcome. Together, these analyses of microarray data sets reveal reduced PTH1R expression is often associated with breast cancer progression and bone metastasis.

### Knockdown of Pth1r in 4T1 cells diminishes responsiveness to intermittent PTH treatment in vivo.

**Figure 4:**
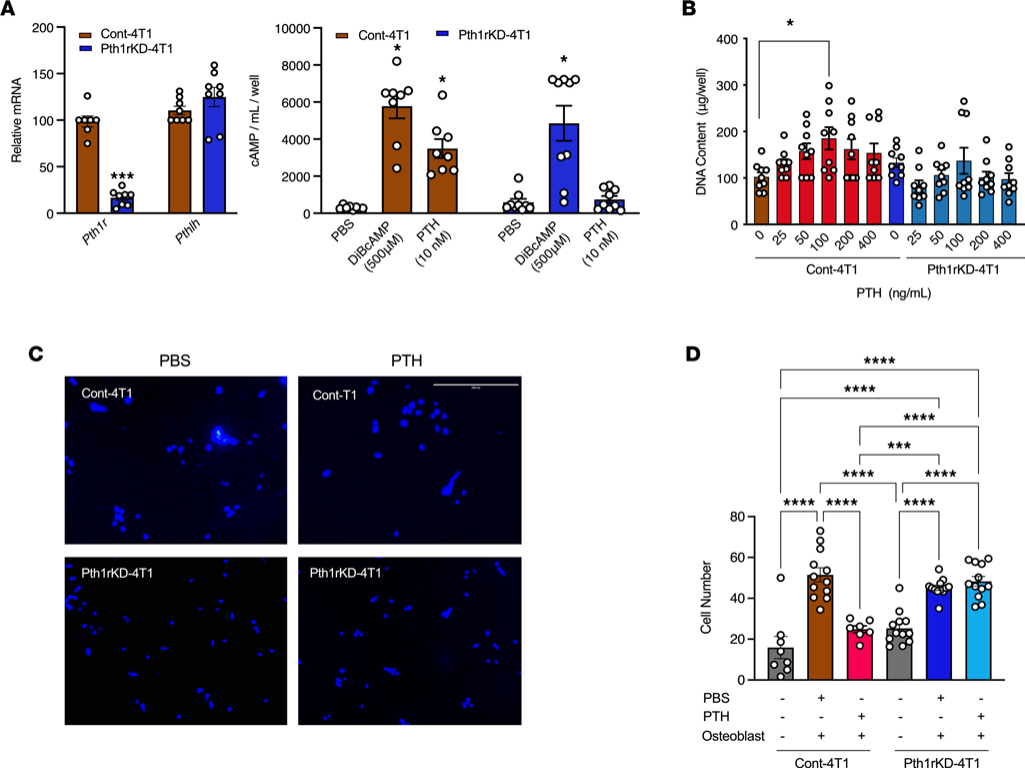
*Pth1r* knockdown in 4T1 cells attenuates the inhibitory effect of PTH on migration toward osteoblasts. (**A**) Relative mRNA expression (*n* = 9) (left panel) and cAMP assay (*n* = 8) (right panel). *P < 0.05 when compared to PBS-treated controls. ***P < 0.001 when compared to Cont-4T1. (**B**) DNA content in Cont-4T1 and Pth1rKD-4T1 cells (*n* = 9). *P < 0.05. (**C** and **D**) Representative images and numbers of Cont-4T1 and Pth1rKD-4T1 breast cancer cells that migrated towards calvarial osteoblasts treated with PTH or PBS in transwell assays (*n* = 12). All values represent mean ± SEM of experiments conducted in triplicate. Statistical significance was evaluated using 2-way ANOVA with Tukey’s test as the post-hoc analysis. ***P < 0.001, ****P < 0.0001. DiBcAMP, dibutyryl cAMP. Scale bar: 200 μm.

To examine the role of PTH1R in breast cancer and bone metastasis, we knocked down *Pth1r* expression in 4T1 cells (Pth1rKD-4T1) by shRNA. *Pth1r* mRNA levels were decreased 86% in Pth1rKD-4T1 cells compared with the LMP vector control cells (Cont-4T1), but *Pthlh* mRNA levels were unchanged (Figure 4A). Basal cAMP levels did not differ between Pth1rKD-4T1 and Cont-4T1 cells. Although a robust increase in cAMP was elicited with a single dose of dibutyryl cAMP (500 μM) in both the Cont-4T1 and Pth1rKD-4T1 cells, PTH treatment (single dose, 10 nM) increased cAMP production in Cont-4T1 but not Pth1rKD-4T1 4T1 cells. PTH had no effect on the proliferation of Cont-4T1 or Pth1rKD-4T1 cells as measured by DNA content (Figure 4B). Migration of Pth1rKD-4T1 breast cancer cells toward calvarial osteoblasts treated with PBS was unchanged in transwell assays compared with the Cont-4T1 cells (Figure 4, C and D). Although PTH treatment reduced the migration of the Cont-4T1 cells toward the calvarial cells, it had no effect on Pth1rKD-4T1 cells. Collectively, the data demonstrate that knockdown of *Pth1r* rendered the Pth1rKD-4T1 cells resistant to the effects of PTH in vitro.

**Figure 5:**
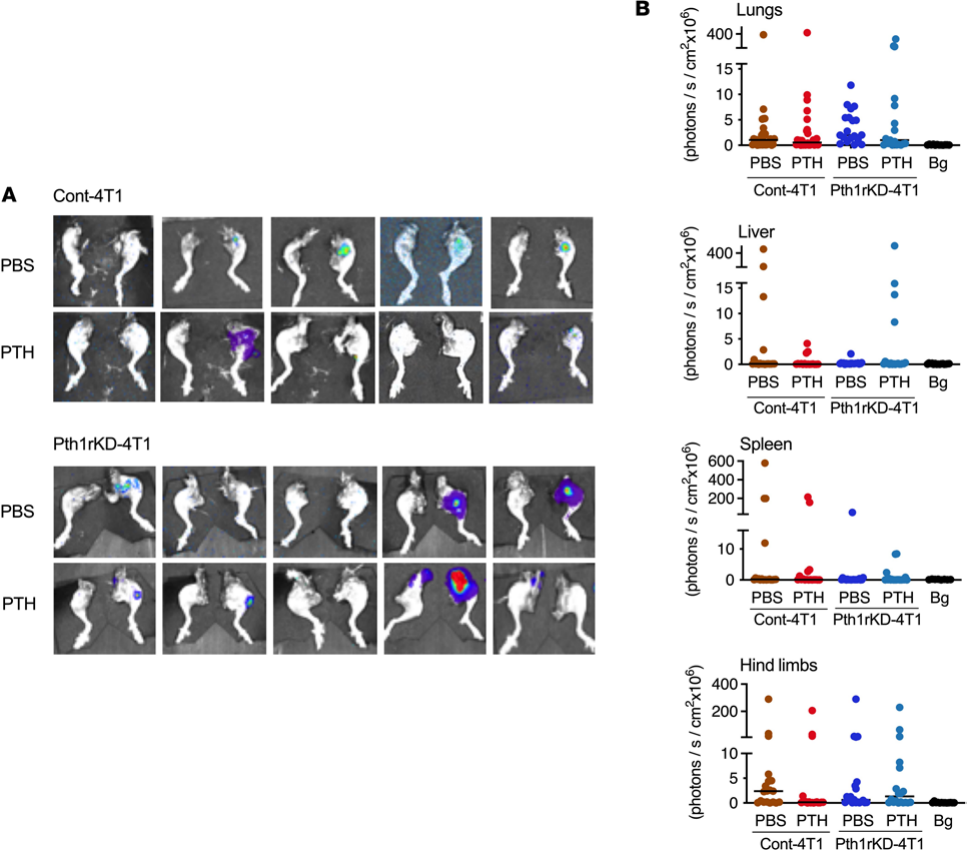
Treatment with intermittent PTH does not reduce skeletal metastasis in orthotopic models of tumors lacking *Pth1r*. (**A**) Representative bioluminescent images of metastases to hind limbs in mice bearing either Cont-4T1 or Pth1rKD-4T1 tumors treated with PBS or intermittent PTH. (**B**) Quantitation of BLI in lungs, liver, spleen, and hind limbs with metastases. All values represent the mean ± SEM of 20 measurements for each group. Statistical significance was evaluated using 2-way ANOVA with Tukey’s test as the post hoc analysis.

To determine whether PTH1R signaling in breast cancer cells is required for bone metastases, intramammary orthotopic tumors of Cont-4T1 or Pth1rKD-4T1 cells were established in 10-week-old female Balb/c mice. Treatment with intermittent PTH (80 μg/d/kg BW administered Monday through Friday) or vehicle (PBS) (Supplemental Figure 3A) was started 24 hours after tumor injection and continued for 4 weeks. BWs were monitored weekly, and no changes were evident with intermittent PTH treatment in mice with Cont-4T1 or Pth1rKD-4T1 tumors compared with their respective PBS-treated control groups (Supplemental Figure 3, B and C). Tumor volumes were equivalent in mice bearing either Cont-4T1 or the Pth1rKD-4T1 tumors (~800 to 900 mm^3^) at the end of 4 weeks by BLI and digital caliper measurements (Supplemental Figure 3, D–F). PTH treatment did not affect growth of either the Cont-4T1 or Pth1rKD-4T1 tumors when compared with PBS treatment. Four weeks after tumor implantation, mice were euthanized, and individual organs underwent BLI to determine the presence of metastasis (Figure 5A and Supplemental Figure 4). Metastases to liver, spleen, and lungs were similar in both number and BLI intensity in all groups of mice (Figure 5B) and did not change with PTH treatment. Bone metastases to hind limbs were also comparable in mice bearing Cont-4T1 or Pth1rKD-4T1 tumors with PBS treatment. However, PTH treatment significantly reduced the incidence of bone metastasis in mice bearing Cont-4T1 tumor but not in mice bearing Pth1rKD-4T1 tumors (4/20 vs 11/20) (Table 2 and Figure 5B). These results confirm that knockdown of *Pth1r* in 4T1 breast cancer cells renders them nonresponsive to the effects of PTH treatment.

### Gene changes with PTH treatment in the tumor–bone microenvironment is abolished with PTH1R knockdown in 4T1 cells.

**Figure 6:**
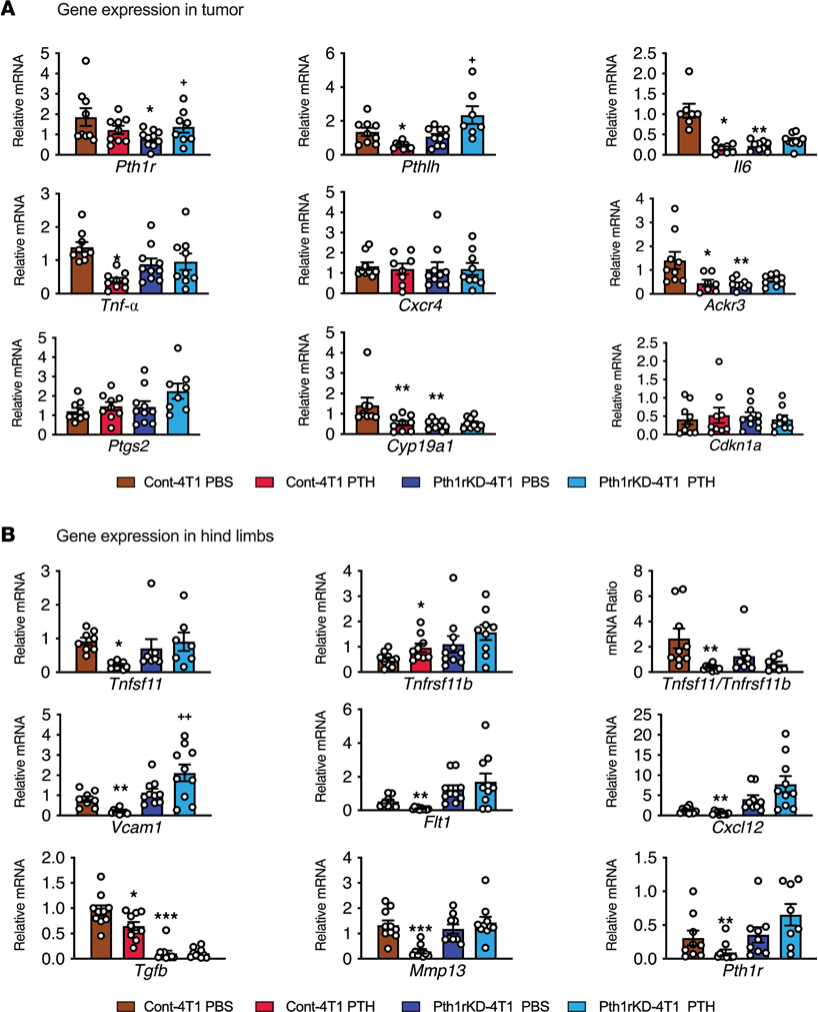
Target gene expression in primary tumors and bone of mice injected with Cont-4T1 and Pth1rKD-4T1 cells. (**A** and **B**)Target gene expression analysis in primary tumors and hind limbs of mice bearing Cont-4T1 and Pth1rKD-4T1 tumors treated with either PBS or intermittent PTH for 4 weeks, using gene-specific primers as described in Methods. All values represent the mean ± SEM of 10 mice for each group. Statistical significance was evaluated using 2-way ANOVA with Tukey’s test as the post hoc analysis. *P < 0.05, **P < 0.01, ***P < 0.001, when compared with PBS-treated Cont-4T1 mice. ^+^*P* < 0.05, ^++^*P* < 0.01 when compared with the Pth1rKD-4T1 PBS group.

Next, we examined the effects of PTH1R knockdown in breast cancer cells on the tumor–bone microenvironment. RNA was isolated from tumors and long bones of tumor-bearing mice (Cont-4T1 or Pth1rKD-4T1) treated with either vehicle or PTH for 4 weeks. Analyses of key target genes involved in the tumor–bone vicious cycle showed similar patterns of gene expression in the vehicle-treated Cont-4T1 and Pth1rKD-4T1 tumors except for *Il6, Ackr3* (encoding CXCR7), and *Cyp19a1*, which were reduced in the Pth1rKD-4T1 cells (Figure 6A). Intermittent PTH treatment reduced the expression of several of these key genes (*Pthlh, Il6, Cyp19a1, Ackr3, and Tnf*) in the Cont-4T1 tumors. These changes, however, were not seen in the Pth1rKD-4T1 tumors treated with intermittent PTH. *Cdkn1A* (encoding the protein p21), a known marker for cell cycle arrest, remained unchanged. It is interesting to note that PTHrP (encoded by *Pthlh*), whose actions are also mediated through the PTH1R, is significantly upregulated in the Pth1rKD-4T1 cells with intermittent PTH treatment. Target gene expression analyses of bones from mice bearing Cont-4T1 tumors showed reciprocal changes in the expression of several genes involved in the tumor–bone vicious cycle (Figure 6B). Significant decreases in the expression of *Tgfb, Mmp13, Flt1*, *Vcam1*, and *Cxcl12* were seen in response to PTH treatment. Additionally, significant reduction in *Tnfsf11* (encoding RANKL) expression accompanied by an increase in *Tnfrsf11b* (encoding OPG) expression resulted in a significant decrease in the RANKL to OPG ratio. Basal expression of various target genes was similar in the mice bearing either the Cont-4T1 or Pth1rKD-4T1 tumors with PBS treatment. PTH treatment, however, did not induce any changes in the expression of various target genes studied except for *Vcam1* in the mice bearing the Pth1rKD-4T1 tumors. These results suggest that the tumor *Pth1r* expression is critical for the actions of PTH in reducing bone metastasis. Knockdown of *Pth1r* in tumors rendered both the tumors and the bone nonresponsive to the actions of intermittent PTH.

### PTHrP is a key mediator of the actions of PTH on breast cancer cell migration.

**Figure 7:**
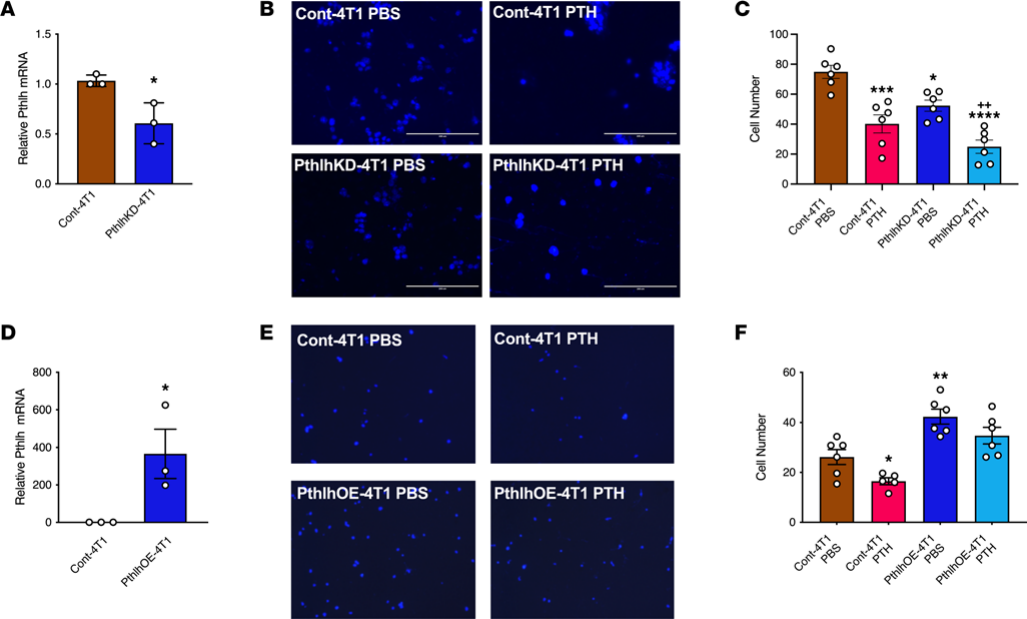
*Pthlh* expression is a key mediator of the actions of PTH on breast cancer cell migration. (**A**) Relative *Pthlh* mRNA levels in 4T1 cells with *Pthlh* knockdown (PthlhKD-4T1) using siRNA. (**B**) Representative images and (**C**) numbers of Cont-4T1 and Pth1rKD-4T1 breast cancer cells that migrated toward MC3T3 osteoblasts treated with PBS or PTH in transwell assays. Scale bar: 200 μm. (**D**) Relative *Pthlh* mRNA levels in PthlhOE-4T1 cells. (**E**) Representative images and (**F**) numbers of Cont-4T1 and PthlhOE-4T1 breast cancer cells that migrated toward MC3T3 osteoblasts treated with PBS or PTH in transwell assays. All values represent the mean ± SEM of at least 6 individual experiments conducted in triplicate. Scale bar: 200 μm. Statistical significance was evaluated using 2-way ANOVA with Tukey’s test as the post hoc analysis. *P < 0.05, **P < 0.01, ***P < 0.001 and ****P < 0.0001 when compared with the PBS-treated control. ^++^*P* < 0.01 when compared with the PTH-treated control.

PTHrP (encoded by *Pthlh)* is a key mediator of the bone–breast vicious cycle, and treatment with intermittent PTH significantly decreased *Pthlh* expression in Cont-4T1 primary tumors while increasing the expression of *Pthlh* in Pth1rKD-4T1 primary tumors (Figure 6A). To determine whether suppression of PTHrP is required for inhibition of 4T1 breast cancer cell migration by PTH treatment, we knocked down *Pthlh* in 4T1 cells (PthlhKD-4T1 cells) using siRNA. Migration toward MC3T3-E1 osteoblastic cells in a transwell system was significantly reduced in PBS-treated PthlhKD-4T1 cells compared with PBS-treated control Cont-4T1 cells. Treatment with intermittent PTH further decreased migration (Figure 7, A–C). Conversely, overexpression of *Pthlh* in 4T1 cells increased migration toward MC3T3-E1 cells and rendered them resistant to the effects of intermittent PTH treatment (Figure 7, D–F). Overall, these results demonstrate that PTHrP is an important mediator of breast cancer cell migration toward the bone and suggest that inhibition of migration by PTH is mediated, at least in part, by suppression of PTHrP expression.

## Discussion

In this study, we establish a critical role for PTH1R in the actions of intermittent PTH in reducing bone metastasis in mouse models of breast cancer. We demonstrate that presence of PTH1R is required in both breast cancer cells and osteoblasts for the inhibitory actions of PTH on bone metastasis, because mice lacking PTH1R in breast cancer cells and mice with impaired PTH1R signaling in osteoblasts both exhibit resistance to the effects of intermittent PTH treatment in reducing bone metastases.

Anabolic actions of PTH on bone remodeling are mediated by PTH1R. PTH1R^OsxKO^ mice exhibit disrupted trabecular bone formation coupled with postnatal growth retardation, extensive defects in growth plate cartilage, and early death (43). We have shown that these defects are accompanied by changes in the expression of target genes within the microenvironment of the bone. Ablation of PTH1R was accompanied by increases in the expression of several genes, such as *Pthlh*, *Flt1*, *Cxcl12*, *Vcam1*, and *Tgfb*. Studies of the bone and its microenvironment have demonstrated that the osteoblast lineage plays unique roles in supporting hematopoiesis and maintaining bone homeostasis. Previous studies from our laboratory have demonstrated that increased of expression of *Vcam1* and *Cxcl12* is implicated in the trafficking of B cells and hematopoietic populations (29, 30) within the bone marrow. VEGF overexpression, acting through VEGF1 and VEGFR2, promotes bone resorption, possibly due to excessive osteoclast resorption (44). Additionally, it has been shown that excessive local production of PTHrP could enhance osteoclast formation and increase bone resorption (45). We previously demonstrated that anabolic PTH treatment reduces osteoclast numbers in the bone (17). Taken together, these reports are consistent with our observation of increased local expression of *Vcam1, Cxcl12, Flt1*, and *Pthlh* in the bones of mice with metastatic breast cancer. Additionally, many of these genes are also associated with the bone–breast cancer vicious cycle, suggesting a possible change in the bone environment conducive for the engraftment of breast cancer cells.

A major impediment for the study of breast cancer metastasis to bone has been the availability of a suitable model that truly represents the metastatic process that occurs in vivo. Syngeneic models, such as the 4T1 cells, have the advantage of spontaneously metastasizing to the bone and also allow for studies to be conducted in mice with normal immune function. The 4T1 model has been validated extensively in the literature (46–48) and is currently 1 of the few models that allows for the study of breast cancer and bone metastasis. To provide a syngeneic background for the 4T1 tumor injections, the PTH1R^OsxKO^ mice were backcrossed to a Balb/c background, which decreased their survival. In the absence of doxycycline, PTH1R^OsxKO^ mice do not survive past 1 month of age (29, 30). Unfortunately, on the Balb/c background, doxycycline-mediated delay of PTH1R deletion until postnatal life did not prolong survival, thus limiting the use of postnatal PTH1R^OsxKO^ mice in our studies. It is not uncommon for *Osx-Cre* transgenic mice to exhibit unexpected or exaggerated phenotypes that could limit their use in in vivo studies (49). Chen et al. (50) showed that *Osx-Cre*, when activated in utero, targets not just osteoblasts but also other cells within the bone marrow in the postnatal mice. *Osx-Cre* activation could occasionally disrupt random off-target genes, giving rise to undesirable phenotypes (33, 51), as seen with our PTH1R^OsxKO^ mice, emphasizing the need for proper controls in studies using these mice. Because G_s_α is a major downstream target for the actions of PTH1R, and we have reported that these mice do not respond to the anabolic actions of PTH (24), we elected to use G_s_α^OsxKO^ mice as an alternative model in our study.

**Figure 8:**
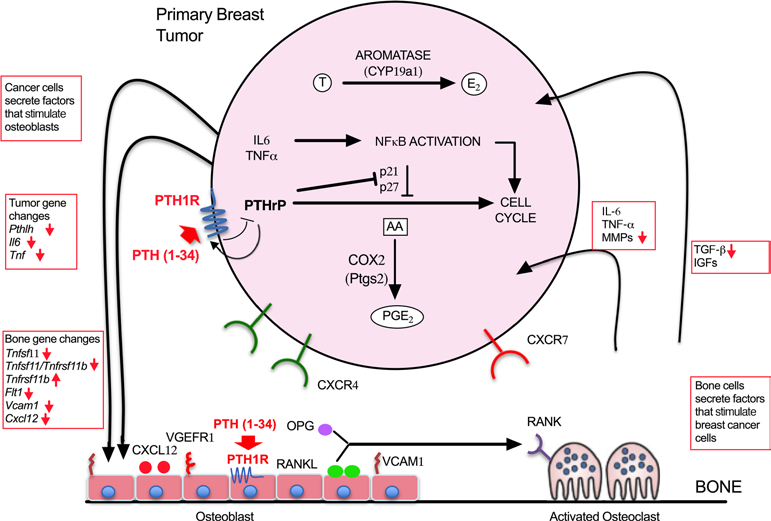
A schematic for the vicious cycle in breast cancer–associated skeletal metastasis. In the mice bearing Cont-4T1 tumors, PTH treatment changes the bone metastatic niche by altering expression of several genes (red arrows) that are implicated in the breast–bone vicious cycle, thereby rendering the bone less favorable to migration of breast cancer to bone. In the mice bearing Pth1rKD-4T1 tumors, knockdown of PTH1R in breast cancer cells perturbs the vicious cycle. In these mice, the response to the actions of intermittent PTH treatment in both the breast cancer and bone are limited, leading to similar rates of skeletal metastasis as seen in the control.

Our proposed model for the role of PTH1R in the pathophysiology of bone metastasis is shown in Figure 8. Disseminated breast cancer cells that migrate to the bone release several factors, such as PTHrP, TGF-β, and IL-6, that perturb the normal bone-remodeling process to create a microenvironment that facilitates tumor engraftment in the bone. Using RNA-Seq analyses, we have shown that conditional ablation of *Pth1r* in osteoblasts resulted in a bone microenvironment where several genes involved in bone metastasis were upregulated. In line with these results, migration of 4T1 breast cancer cells toward calvarial osteoblasts from PTH1R^OsxKO^ and G_s_α^OsxKO^ mice was enhanced when compared with control mice. In vivo experiments in G_s_α^OsxKO^ mice bearing 4T1 breast cancer tumors, however, showed comparable skeletal metastasis in both control and G_s_α^OsxKO^ mice. This discrepancy could be due to greater heterogeneity within the tumor in vivo, which may alter the tumor microenvironment as well as the migratory potential of the cells. It is also likely that other cell populations in the bone microenvironment play a role in skeletal metastasis.

Disruption of PTH1R signaling abolished the inhibitory effects of PTH, and the incidence of bone metastases was similar in both PTH- and PBS-treated G_s_α^OsxKO^ mice. Likewise, knockdown of *Pth1r* in 4T1 cells did not alter the incidence of bone metastasis but rendered the cells nonresponsive to the inhibitory actions of PTH treatment both in vitro and in vivo. These results suggest that the absence of PTH1R signaling in either osteoblasts or breast cancer cells alone was not sufficient to affect the ability of the cancer cells to disseminate and metastasize to the bone. The presence of *Pth1r*, however, was critical in both the tumor and the bone for the inhibitory actions of PTH on skeletal metastasis. PTH, acting through PTH1R, plays a key role in the bone–breast vicious cycle, altering several key genes implicated in bone metastases and rendering the niche less favorable to the homing of breast cancer cells. These inhibitory effects were lost when PTH1R signaling was perturbed either in tumor or bone.

PTH1R is the common receptor for the actions of both PTH and PTHrP. It is expressed abundantly in the classical target tissues of PTH action, including kidney and bone, and is co-expressed with PTHrP in other malignant and nonmalignant tissues. In contrast to the substantial body of evidence linking PTHrP to breast cancer progression and metastasis (52, 53), much less is known about PTH1R and its role in cancer metastasis. Recent studies have shown distinct immunostaining for PTH1R in many types of cancers, such as breast, colorectal, and prostate cancers; osteosarcomas; and renal and gastric carcinomas (54). Our results, based on analyses of available microarray data sets, demonstrated that expression levels of *Pth1r* in primary tumors were significantly lower than in healthy breast tissue and that tumor progression was often accompanied by a significant reduction in *Pth1r* levels. When metastatic tumors were compared with primary tumors, 1 study showed significant reduction in *Pth1r* levels, and there was no change in a second study (39, 42). The few published studies in breast tumors have reported (55), however, that *Pth1r* expression levels were higher in metastatic tumors than in primary tumors in patients with breast cancer and suggested that overexpression of the receptor was the driver for the autocrine actions of PTHrP in bone metastasis. These studies, however, did not look for the expression of *Pth1r* in healthy breast tissue and did not account for the possibility of nonreceptor-mediated actions of PTHrP. Overall, these results suggest a conflicting role for the presence or absence of the PTH1R in cancer and metastasis, but its presence is critically important for the biological actions PTH in changing the tumor microenvironment.

PTHrP has a key role in breast cancer progression and metastasis (5). Like PTH, the actions of PTHrP are also mediated through PTH1R. Studies have shown that PTHrP and PTH1R are co-expressed in many invasive breast carcinomas (20, 22). Published studies suggest that PTHrP inhibits tumor progression in early stages of disease, while functioning in the opposite manner to promote tumor development and metastasis in advanced cancers. This is especially true in bone metastasis, where PTHrP-mediated osteolysis is critical for tumor cells to establish as colonies and grow. We have shown previously that treatment of 4T1 tumors with intermittent PTH downregulates the expression of PTHrP along with other cytokines and growth factors, thereby altering the breast–bone vicious cycle. Manipulation of endogenous *Pthlh* expression using siRNA reduced the migratory potential of 4T1 cells toward osteoblasts in vitro, and this potential was further reduced by PTH. Likewise, the effects of PTH were blunted in 4T1 cells overexpressing *Pthlh*. In advanced cancers, such as those caused by 4T1 breast cancer cells, PTH suppresses the expression of *Pthlh* in tumors, slowing the tumor–bone vicious cycle and leading to reduced incidence of bone metastases. Taken together, these results identify yet another important regulator for the antimetastatic actions of PTH.

In conclusion, we demonstrate, using transgenic models and shRNA knockdown, that PTH1R signaling is critical in both breast cancer cells and osteoblasts for the inhibitory actions of PTH treatment on bone metastases. The effects of PTH on reducing bone metastasis were mediated, in part, by PTHrP. Collectively, these data suggest an interplay of events among PTH1R, PTH, and PTHrP, particularly in breast cancer–mediated skeletal metastasis, and offer new directions for research.

## Methods

### In vivo studies

#### Study approval.

All animal procedures were performed in compliance with the guidelines approved by Stanford University Administrative Panels on Laboratory Animal Care.

#### Mice.

Mice lacking *Pth1r* or G_s_α in osteoprogenitors were generated as previously described (32, 56, 57). Postnatal deletion of G_s_α was achieved by administration of 100 μg/mL doxycycline in drinking water from conception until weaning (day 21). Genotyping was performed on tail genomic DNA as previously described (24, 31, 32). Because the transgenic mice were of mixed genetic background, they were backcrossed into the Balb/c background for 5 generations before use in this study. Cre^–^ PTH1R^fl/fl^ or G_s_α^fl/fl^ mice were routinely used as controls unless otherwise specified. For experiments that did not require transgenic mice, female Balb/c mice (Jackson Laboratories) were used. All mice were approximately 10 weeks old at the start of the experiment. Mice were housed in a designated pathogen-free facility and fed irradiated mouse chow and autoclaved water throughout the experimental period.

#### Mammary fat pad injections.

Approximately 1 × 10^6^ murine 4T1 breast cancer cells suspended in 100 μL of culture medium–Matrigel mixture (1:1) were injected into the fourth left mammary fat pad of syngeneic mice (Balb/c or transgenic mice backcrossed to Balb/c background) approximately 10 weeks old to establish orthotopic xenografts. At 24 hours after tumor injection, mice were randomized to receive PTH(1-34) (Bachem) 80 μg/kg/d Monday through Friday or PBS for 4 weeks. BWs and tumor volumes were measured weekly, and tumor volumes were calculated from 2 tumor measurements using the following formula: tumor volume = (length × width^2^)/2 (58). After 4 weeks, animals were euthanized, and individual organs were harvested for BLI.

#### BLI.

BLI was performed on live animals under isoflurane anesthesia, using a Lago Optical imager from Spectral Instruments Imaging after injection of luciferin substrate (3.33 mg/mouse). Luciferin substrate was injected into the mice 10 minutes prior to euthanasia. Average radiance was measured and quantified for all organs using the Aura Image Software provided by the vendor.

#### Histology.

Hind limbs were fixed in 4% paraformaldehyde for 48 hours, decalcified in 10% EDTA for 21 days, then processed and embedded in paraffin. Serial sections of 5 μM thickness were cut, H&E stained, and visualized for the presence of metastasis.

### In vitro studies

#### Cell culture.

Murine 4T1 breast cancer cells obtained from ATCC were stably transfected to express the firefly luciferase and the enhanced GFP using the Sleeping Beauty transposon plasmid pKT2/LuBiG to generate the 4T1-/LuBiG (4T1) clones (a gift from Michael Bachmann, Stanford University, Stanford, CA) (59, 60). 4T1 cells were submitted to Genetica and were validated to be free of any cross-contamination from human DNA. Cells were cultured in DMEM:F12 media supplemented with 10% FBS, 100 IU/mL penicillin, and 100 μg/mL streptomycin, and maintained at 37ºC with 5% CO_2_ in a humidified incubator. Blasticidin (0.5 μg/mL medium) was used to maintain selection pressure.

#### Knockdown of Pth1r in 4T1 murine breast cancer cells.

To stably silence *Pth1r* expression 4T1 cells, MiR30-based shRNA was designed and synthesized using GeneArt and cloned into the LMP vector as described previously (61, 62). In this study, we used the Pth1r1.358 shRNA construct (a gift from Carl Walkley, Fitzroy, Victoria, Australia) to knockdown the expression of *Pth1r* in 4T1 cells. Briefly, subconfluent cultures of Phoenix cells (63) in 10 cm dishes were transfected with 10 μg of either the LMP control or the Pth1r1.358 shRNA constructs using Lipofectamine to generate retroviral supernatants. 4T1 cultures were infected with either the LMP control or the Pth1r1.358 viral supernatant. Transfected cells were selected using 2 μg/mL puromycin to generate Cont-4T1 or Pth1rKD-4T1 cells. Cells were screened for the expression of *Pth1r* mRNA and their ability to reduce cAMP levels in response to PTH treatment, and a pool of stably transfected cells was used in the animal studies to generate primary tumors.

#### cAMP assay.

Cells expressing the shRNA were tested for *Pth1r* expression by measuring the cAMP response to 10 nM PTH using the cAMP ELISA kit from Cayman Chemicals according to the manufacturer’s instructions.

#### Cell proliferation assay.

Cont-4T1 or Pth1rKD-4T1 cells were seeded (100,000 cells/well) in 6-well culture plates in DMEM:F12 medium containing 10% FBS and treated with graded concentrations of PTH for 6 days, as described previously (64). DNA content was assessed at the end of the treatment period (65) and used as a measure of cell proliferation.

#### Isolation of calvarial cells.

Primary osteoblasts were isolated from neonatal calvariae of both PTH1R^OsxKO^ and G_s_α^OsxKO^ mice by serial collagenase digestion, as previously described (66). The isolated osteoblasts were then propagated in αMEM medium containing 10% FBS and used in the migration assays described below.

#### Migration assays.

MC3T3-E1 pre-osteoblastic cells or primary osteoblastic cells harvested from neonatal calvariae of either PTH1R^OsxKO^ or G_s_α^OsxKO^ mice and littermate controls (32) were seeded (2 × 10^4^ cells/well) in the lower chamber of the 8 μm pore polycarbonate Transwell Permeable Support (Costar) system and allowed to attach overnight. Cells were then treated with either PBS (control) or PTH (50 ng/mL) and incubated for 6 hours. Medium was changed at the end of 6 hours and cells were incubated overnight with fresh medium. Simultaneously, 5 × 10^4^ Cont-4T1 or Pth1rKD-4T1 breast cancer cells were seeded in the upper inset of the transwell setup in serum-free medium. After overnight incubation, the upper chamber was transferred to the transwell setup and cells were allowed to migrate for 4 hours. At the end of incubation period, cells that migrated to the underside of the membrane were fixed and stained with DAPI. Five random fields per membrane were photographed using a fluorescence microscope, and total numbers of cells were counted using Image J, version 1.48, software (NIH).

### RNA-Seq analysis

Total RNA was extracted from sorted cells using the RNeasy Plus Micro Kit (Qiagen) (29). Libraries were then prepared with the SMART-Seq v4 Ultra Low Input RNA Kit for Sequencing and SMARTer ThruPLEX DNA-Seq Kit (Takara Bio). Sequencing was performed by the Illumina HiSeq 4000 (Genome Sequencing Service Center at the Stanford Center for Genomics and Personalized Medicine). Sequencing reads were aligned with STAR and analyzed by DESeq2. Gene ontology analysis was performed at Gene Ontology Consortium. All transcriptome raw data are publicly available in the NCBI Gene Expression Omnibus (GEO; accession no. GSE185944).

### RNA isolation and real-time PCR

Total RNA was isolated from primary osteoblasts (obtained by serial collagenase digestion on flushed long bones) and primary tumors after homogenization using the Trizol reagent (Invitrogen) according to the manufacturer’s instructions. RNA (1.5 μg) was subjected to reverse transcription using the iScript RT kit (Bio-Rad), and gene expression was determined by real-time PCR using the CFX96 real-time PCR detection system (Bio-Rad) with gene-specific primers (17) and the SYBR green qPCR kit (Bio-Rad). Target gene expressions were normalized to β-actin and the relative changes in mRNA levels were assessed by the comparative Ct method (67).

### Microarray analysis

To study the expression of PTH1R in breast cancer tumors, we identified publicly available microarray data sets from the NCBI and GEO data repositories. The following keywords were used to search the data repositories: breast cancer, bone metastasis, human, murine, gene expression profiling by array. Data sets were further filtered to exclude studies that included drug treatments and metastasis to organs other than bone. Thirty data sets were found, of which 10 studies (*n* = 3 murine and 7 human) fit the required criteria. They included GDS4077, GDS5666, GSE21444, GDS4091, GSE137842, GDS3853, GSE8977, GSE9014, GDS4761, and GSE57947. All data sets were on an Affymetrix GeneChip Human Genome U133 Plus 2.0 or Affymetrix Human Gene 1.1 platform. Data analysis and statistical analysis were conducted using GEO2R (an online data analysis tool used to analyze GEO data sets under the same experimental conditions), and raw data were retrieved from individual data files as bar charts.

### Transient transfections for overexpression and knockdown of Pthlh

Mouse *Pthlh*-tagged ORF mammalian expression plasmid in a pCMV3 vector was obtained from Origene, and the siGENOME siRNA targeted toward the mouse *Pthlh*/PTHrP was obtained from Dharmacon. We transiently transfected 4T1 cells with either the mouse *Pthlh* expression plasmid to overexpress *Pthlh* (PthlhOE-4T1) or siRNA to knockdown *Pthlh* (PthlhKD-4T1), per the manufacturer’s instructions. Knockdown and/or overexpression of *Pthlh* was confirmed using gene-specific primers. Empty vectors were used as negative controls for the expression of *Pthlh*. Transiently transfected cells were treated with PTH for 6 hours, as described above. MC3T3-E1 cells were seeded in the lower chamber of the transwell migration setup, and migration studies were carried out as described above.

### Statistics

Statistical analyses were performed using the GraphPad Prism 5 software (GraphPad Software). *Z* proportion scores were calculated where applicable, and data were evaluated using ANOVA with either multiple comparisons or 1-way ANOVA with Tukey’s test as the post hoc analysis. All data are shown either as individual data points or expressed as mean ± SEM, unless otherwise indicated.

## Author contributions

SS and JWY contributed to the study conception and design; development of the methodology; analysis and interpretation of data; writing, review, and revision of manuscript; administrative, technical, and material support; and study supervision. SS, AN, TK, HZ, MJK, and VN contributed to data acquisition.

## Supplementary Material

Supplemental data

## Figures and Tables

**Table 1:**
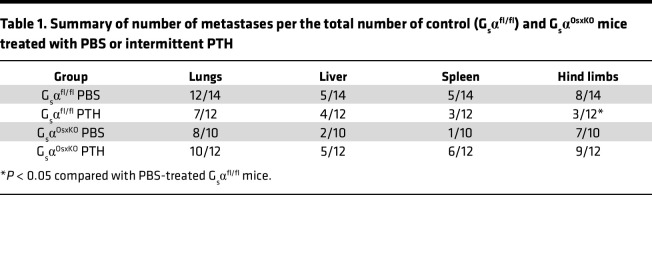
Summary of number of metastases per the total number of control (G_s_α^fl/fl^) and G_s_α^OsxKO^ mice treated with PBS or intermittent PTH

**Table 2:**
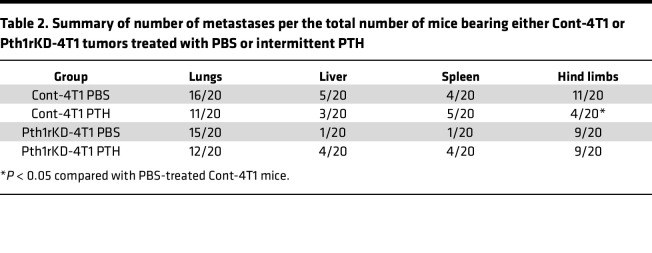
Summary of number of metastases per the total number of mice bearing either Cont-4T1 or Pth1rKD-4T1 tumors treated with PBS or intermittent PTH

## Version 1 01/24/2023

In-Press Preview

## Version 2 03/08/2023

Electronic publication
