# Supplementary material for: Parathyroid hormone 1 receptor signaling mediates breast cancer metastasis to bone in mice
Source: JCI Insight. 2023 Mar 8;8(5):e157390. doi: 10.1172/jci.insight.157390 (PMC10077472; doi:10.1172/jci.insight.157390)
Supplement: Supplemental data [file jciinsight-8-157390-s176.pdf]

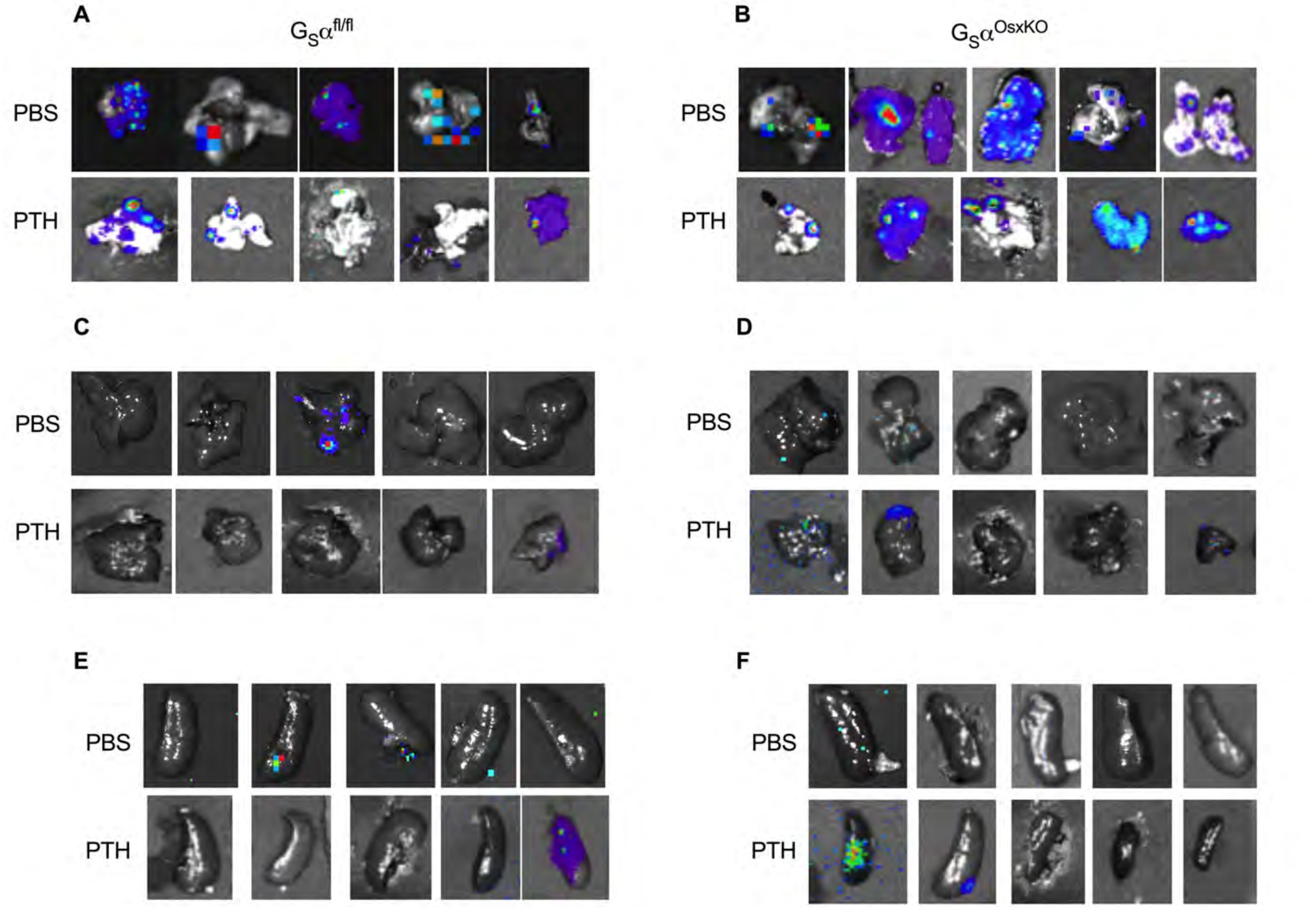

SUPPLEMENTARY FIGURE 1: Representative bioluminescence images in distal organs of $G_S\alpha^{fl/fl}$ and $Gs\alpha^{OsxKO}$ mice bearing 4T1 tumors.

Bioluminescence (BLI) images in distal organs of $G_S\alpha^{fl/fl}$ (left panels) and $G_S\alpha^{OsxKO}$ (right panels) mice bearing 4T1 tumors (n = 10) treated with either PBS or PTH. Representative images **(A, B)** lungs, **(C, D)** liver, and **(E, F)** spleen.

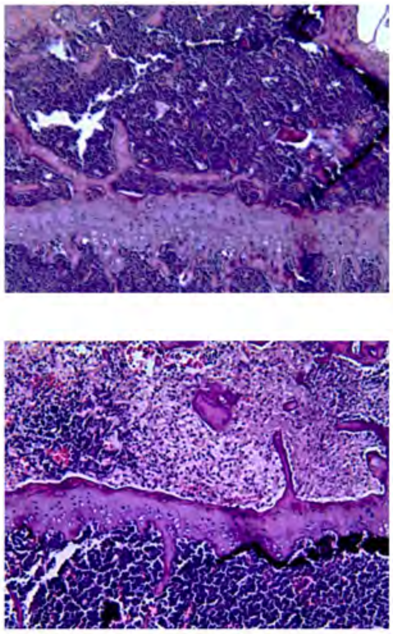

$G_s\alpha^{OsxKO}$
no metastasis

$G_s\alpha^{OsxKO}$
with metastasis

Supplementary Figure 2: Representative H&E-stained images of tibia from of $G_s\alpha^{OsxKO}$ mice with and without metastasis (20x magnification).

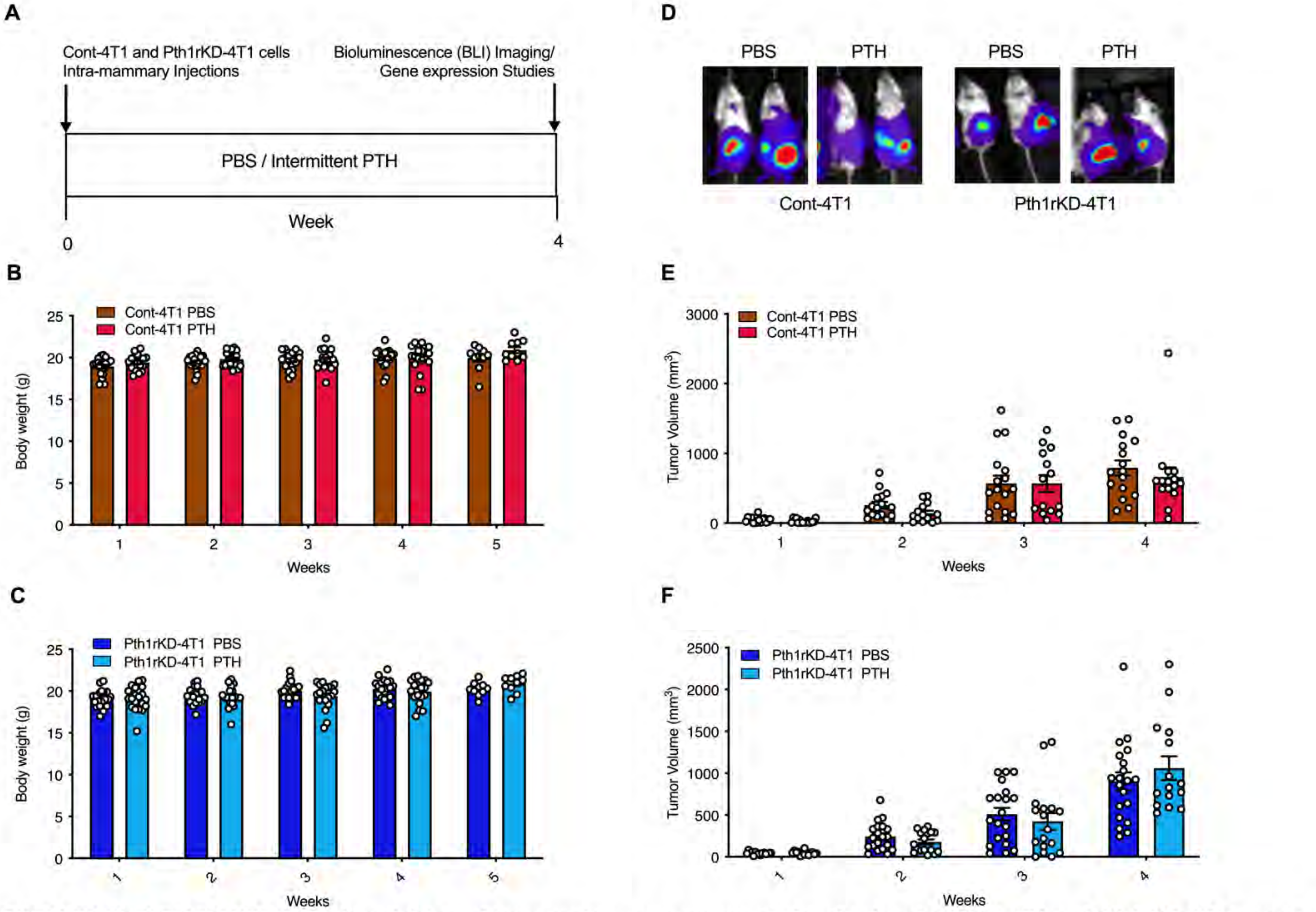

SUPPLEMENTARY FIGURE 3: Body weights and tumor volume measurements in mice bearing Cont-4T1 and Pth1rKD-4T1 tumors **(A)** Experimental Design, **(B and C)** weekly body weight measurements, and **(D)** representative endpoint bioluminescence (BLI) images of Balb/c mice bearing either Cont-4T1 or Pth1rKD-4T1 tumors treated with either PBS or PTH. **(E and F)** Weekly tumor measurements. All values represent Mean ± SEM of n = 20 for each group. Comparisons were made to PBS-treated controls in each group for statistical analyses.

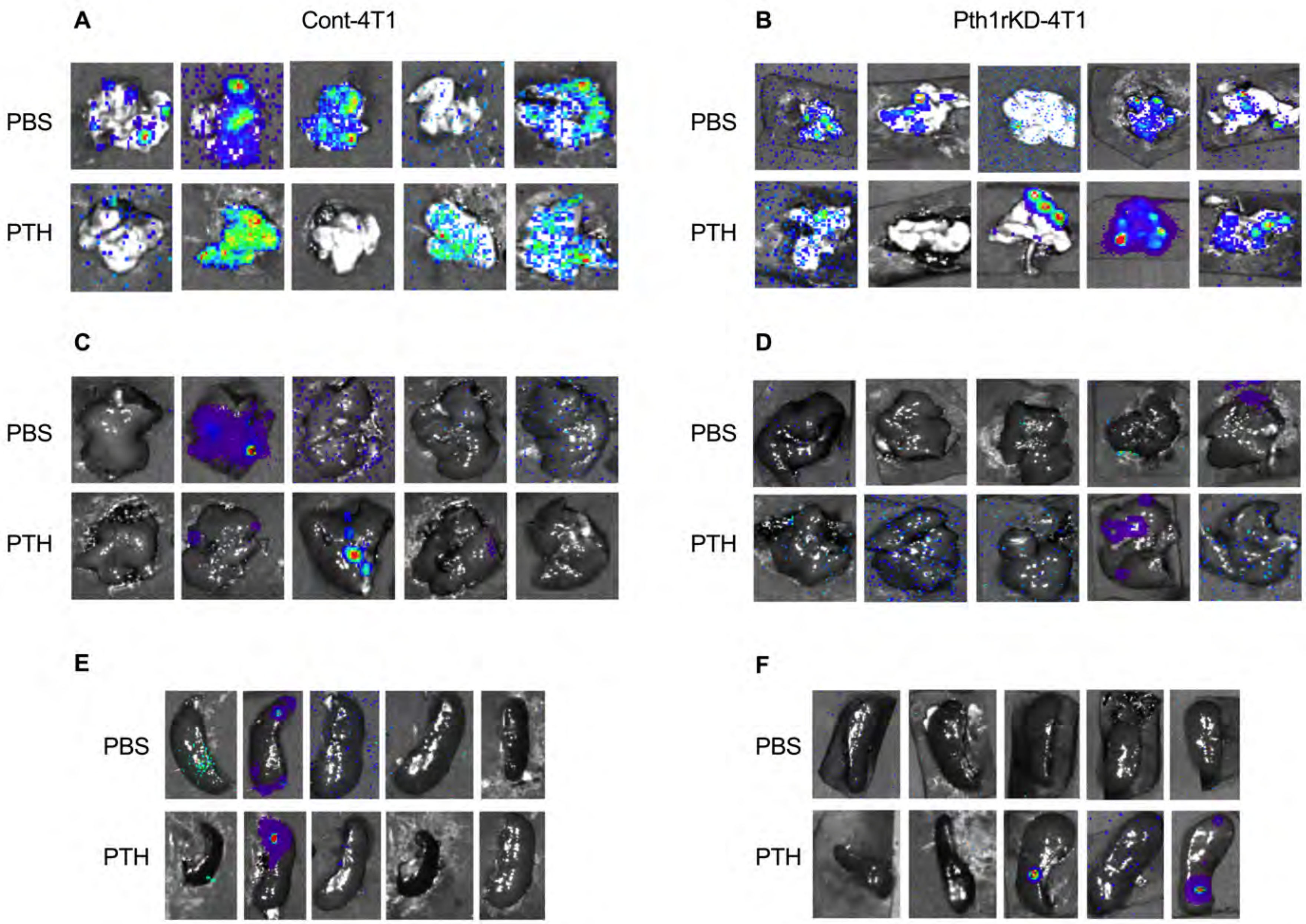

SUPPLEMENTARY FIGURE 4: Representative bioluminescence images in distal organs of Balb/c mice bearing Cont- 4T1 or Pth1rKD-4T1 tumors

Bioluminescence (BLI) images of distal organs from Balb/c mice bearing Cont-4T1 (left panels) or Pth1rKD-4T1 (right panels) tumors (n = 20) treated with either PBS or PTH. Representative images **(A and B)** lungs **(C and D)** liver and **(E and F)** spleen.
